# The protective role of serum uric acid against premature membrane rupture in gestational diabetes: a cross-sectional study

**DOI:** 10.1186/s12902-021-00736-3

**Published:** 2021-05-06

**Authors:** Meixiang Guo, Jun Lu, Xuemei Yu, Xiaowen Hu, Wenjing Hou, Shuguang Pang

**Affiliations:** 1 Department of Endocrinology and Metabolism, Institute/University/Hospital: Jinan Central Hospital, Cheeloo College of Medicine, Shandong University, No. 105 Jiefang Road, Jinan City, 250013 Shandong Province China; 2 Departments of Endocrinology and Metabolism, Fengxian District Central Hospital, Shanghai, China; 3 Department of Endocrinology and Metabolism, Putuo Hospital, Shanghai University of Traditional Chinese Medicine, Shanghai, China; 4 Departments of Obstetrics and Gynaecology, Fengxian District Central Hospital, Shanghai, China; 5 Department of Endocrinology Jinan Central Hospital Affiliated to Shandong First Medical University, Jinan, China

**Keywords:** Uric acid, Premature rupture of foetal membranes, Gestational diabetes

## Abstract

**Background:**

Uric acid has strong antioxidant activity, whereas its oxidative damage is closely related to many diseases. We assessed the association between serum uric acid (SUA) levels and premature rupture of membranes (PROM) in pregnant women with gestational diabetes (GDM) in China.

**Methods:**

In this cross-sectional study, a total of 456 pregnant women were enrolled. Anthropometric parameters for pregnant women were collected within 12 weeks of gestation. Weight gain during pregnancy was obtained from the patients’ records. GDM was diagnosed according to 75-g oral glucose tolerance tests at the 24-28th week of gestation, and SUA was determined simultaneously. PROM was identified as the natural rupture of foetal membranes before the first stage of labour. Logistic models were fitted to identify the presence of PROM using clinical characteristics with (Model 2) or without serum uric acid (Model 1).

**Results:**

There were differences in BMI, haemoglobin A1c, fasting blood glucose, 1-h postprandial glucose (PG), 2-h PG, insulin levels, triglycerides,weight gain during pregnancy, the rate of macrosomia, fetus birth weight and PROM between women with and without GDM (all *P* < 0.05). Furthermore, GDM women with PROM had lower levels of SUA compared to those without PROM (*P* = 0.030). The odds ratio of PROM decreased with increasing SUA levels. The area under the receiver operating characteristic curves for PROM based on Model 2 was larger than that in Model 1 (0.86 versus 0.71, *P* < 0.05).

**Conclusion:**

Relatively elevated SUA levels at the 24-28th weeks of gestation were associated with a lower risk of PROM in women with GDM. Therefore, SUA may be a protective factor for PROM in GDM patients. The optimal concentration of uric acid in different diseases and different populations needs to be further studied.

**Supplementary Information:**

The online version contains supplementary material available at 10.1186/s12902-021-00736-3.

## Background

Premature rupture of the membranes (PROM) happens when foetal membranes rupture during pregnancy at any time before the onset of uterine contractions and occurs in approximately 5 to 10% of pregnancies. PROM that occurs prior to 37 weeks of gestation is referred to as preterm PROM [1]. PROM is not only a common obstetrics complication but also the leading cause of perinatal mortality and morbidity [2]. PROM is the major determinant of newborn survival and length of stay in the neonatal intensive care unit [3]. Additionally, this complication is a common reason for sub-par health in neonates [4], and harmful to newborn health in many ways [5]. PROM is also one of the most common maternal complications in women with gestational diabetes (GDM) [2].

GDM affects approximately 6% of pregnant women [6]. The International Association of Diabetes and Pregnancy Study Groups (IADPSG) defines GDM as “any degree of glucose intolerance with onset or first recognition during pregnancy” [7]. After a new family planning policy was put into effect in China, the number of women with advanced maternal age increased, which had an influence on the number of women with GDM; the incidence of GDM increased, as did the risk of adverse perinatal outcomes [8]. An important aspect of GDM is increased levels of oxidative stress (OS) biomarkers. A study suggested that OS may affect the pregnancy outcome of GDM patients [9]. Placental histological examinations have indicated that OS also plays an important role in inducing inflammation and impairing perfusion in the placenta [10]. OS-induced damage affects not only the intrauterine tissues and foetal membranes of the placenta but also all major cellular factors of foetal cells; this damage promotes foetal cell ageing [11]. After receiving a signal for disease or normal maturation, the foetus produces collagenase V, which destroys the basement membrane and foetal membrane function [12]. It is not clear whether factors related to OS have a relationship to PROM in women with GDM. Although PROM is common in women with GDM, to the best of our knowledge, no studies have identified metabolic risk factors for PROM in women with GDM. Therefore, to address this question, we studied the morbidity of PROM in GDM and non-GDM pregnancies and explored whether metabolic risk factors would have any effect on PROM in the GDM group.

## Methods

### Subjects and grouping

In this cross-sectional study, subjects were recruited from the maternity clinic of the Central Hospital of Shanghai Fengxian District from November 2016 to January 2019. The patients were included according to the following criteria: 1) 20 to 40 years old, 2) had a pregnancy examination in our hospital, 3) were prepared to complete a 75-g oral glucose tolerance test (OGTT) screening at 24-28th weeks of gestation and 4) were to give birth in our hospital to ensure the integrity of the data. All subjects underwent a medical history evaluation, and those with the following conditions were excluded: 1) a history of smoking (*n* = 1), 2) diabetes diagnosis before pregnancy (*n* = 15), 3) polycystic ovary syndrome (n = 15), 4) hypertension (*n* = 8), 5) liver disease (*n* = 3), or 6) drugs that may affect glucose metabolism (*n* = 5). Finally, a total of 152 women with GDM and 304 individuals without GDM were included in this study.

### Data collection

Pre-pregnancy BMI (kg/m^2^) was calculated on the basis of self-reported weight measurements before the pregnancy.

Venous blood samples were drawn after a minimum of a 10-h overnight fast during 24-28th gestational weeks. Serum biochemical parameters were measured using a Beckman DXC800 automatic biochemical analyser. Haemoglobin A1C (HbA1c) levels were measured using high-performance liquid chromatography (TOSOH HLC-723 G7, Japan), and insulin levels were determined using an insulin detection kit and its reagent (Roche Diagnostics, Germany).

One-step 75 g OGTTs were performed during 24-28th gestational weeks. Fasting and postload blood glucose and insulin levels were measured at 0, 60, and 120 min. GDM was diagnosed according to the 2013 World Health Organization diagnostic criteria: fasting blood glucose ≥5.1 mmol/L,1-h OGTT blood glucose ≥10.0 mmol/L, or 2-h OGTT blood glucose ≥8.5 mmol/L.

All participants were inpatients before delivery. Weight gain during pregnancy was collected, and primary adverse outcomes were confirmed by experienced obstetricians. The natural rupture of foetal membranes before the first stage of labour was diagnosed as PROM. All subjects had delivered and finished follow-up by December 2019.

### Statistical analysis

Continuous data were tested for normal distribution using the Kolmogorov-Smirnov test. Normally distributed continuous data are presented as the Means ± standard deviation, and a t-test was used for comparison. Nonnormally distributed continuous data are presented as medians (range), and a Mann-Whitney U test was used for comparison. Categorical variables are expressed as frequencies (percentages), and a chi-square test was used for comparison. The blood uric acid level of the total population was divided into three groups according to the tertiles. The association between serum uric acid (SUA) level and PROM was assessed using binary logistic regression, and odds ratios (ORs) and 95% confidence intervals (95% CIs) are presented. The predictive value of different models for PROM was analysed using receiver operating characteristic (ROC) analysis. Statistical analyses were performed using SPSS for Windows version 20.0 (IBM SPSS, New York, United States). Two-sided *P*-values < 0.05 were considered statistically significant.

## Results

### Comparison of clinical characteristics and outcomes

Table 1 lists the clinical characteristics of the patients involved in this study. There were differences in the levels of HBA1c, fasting plasma glucose (FPG), 1-h postprandial glucose (PG), 2-h PG, 2-h postprandial insulin (2-h INS), HOMA-IR, HOMA-β, white blood cells, BMI, fasting insulin (FIN), 1-h postprandial insulin (1-h INS) and triglycerides between the non-GDM and GDM groups (all *P* < 0.05). Moreover, there were significant differences in weight gain during pregnancy (*P* = 0.014), foetal birth weight (*P* = 0.003), rate of PROM (*P* = 0.033) and macrosomia (*P* = 0.004) between the two groups. There was a similar level of systolic blood pressure before delivery in the two groups.

**Table 1:** Clinical characteristics and main outcome in the non-GDM and GDM groups

Variable	P-NGT group	GDM group	*P*-value
	(*n* = 304)	(*n* = 152)	
Age (years)	29 (26, 32)	29 (26, 33)	0.171
Pre-pregnancy BMI (kg/m^2^)	21.23 (19.56, 23.24)	22.32 (20.20, 24.17)	0.001*
Systolic blood pressure (mmHg)	110 (104, 120)	112 (102, 120)	0.294
Diastolic blood pressure (mmHg)	70 (65, 75)	70 (65, 78)	0.673
HbA1c (%)	4.80 (4.50, 4.90)	4.90 (4.40, 5.20)	< 0.001**
FPG (mmol/L)	4.20 (4.00, 4.50)	4.90 (4.40, 5.65)	< 0.001**
1-h PG (mmol/L)	6.70 (5.70, 7.95)	9.80 (7.78, 10.90)	< 0.001**
2-h PG (mmol/L)	6.30 (5.40, 6.90)	8.50 (6.13, 9.20)	< 0.001**
FINS (mU/L)	8.72 (5.96, 12.98)	10.26 (6.69, 15.46)	0.010*
1-h INS (mU/L)	61.49 (42.55, 86.39)	74.00 (48.65, 117.00)	0.010*
2-h INS (mU/L)	60.70 (39.81, 82.71)	84.06 (51.94, 133.75)	< 0.001**
HOMA-IR	1.64 (1.09, 2.51)	2.20 (1.46, 3.84)	< 0.001**
HOM-AB	3.68 (1.73, 3.68)	1.75 (0.94, 2.56)	< 0.001**
ALT (U/L)	15 (11, 23)	13 (10.00, 22)	0.301
Total bilirubin (μmol/L)	8.00 (6.90,10.00)	8.00 (6.25,9.69)	0.569
Creatinine (umol/L)	46 (43, 51)	47 (42, 51)	0.494
Uric acid (μmol/L)	213 (184,259)	230 (195,256)	0.130
Triglycerides (mmol/L)	2.17 (1.73,2.80)	2.41 (1.90,3.11)	0.023*
Total cholesterol (mmol/L)	6.07 (5.53, 6.82)	5.97 (5.27, 6.66)	0.093
White blood cell (× 10^9^/L)	9.12 ± 2.02	9.90 ± 2.07	< 0.001*
Platelet count (×10^9^/L)	217.50 ± 48.21	224.07 ± 42.06	0.061
Parity	2 (1,2)	1 (1,2)	0.439
Weight gain during pregnancy (Kg) Systolic pressure before delivery (mmHg)	15.85 ± 5.37 120 (110,126)	14.09 ± 6.47 120 (117,127)	0.014* 0.135
Fetus birth weight (g)	3398.92 ± 461.79	3542.10 ± 448.09	0.003*
PROM (n,%)	22 (7.6)	19 (13.29)	0.033*
Macrosomia (n,%)	20 (6.99)	24 (16.78)	0.004*
Fetal distress in the uterus (n,%)	25 (8.74)	13 (0.091)	0.991

Data represent the Mean and standard deviation, medians (interquartile ranges) or percentages (%). Significant at *P* < 0.05. *P* < 0.05*, *P* < 0.001**, Calculated using the Mann-Whitney U test or Chi-square test

*PROM* premature rupture of the membranes*, BMI* body mass index*, HbA1c* hemoglobin*, FPG* fasting plasma glucose*, 1-h PG* 1-hour postprandial glucose*, 2-h PG* 2-hour postprandial glucose*, FINS* fasting insulin*, 1-h INS* 1-hour postprandial insulin*, 2-h INS* 2-hour postprandial insulin*, ALT* alanine aminotransferase*, Macrosomia* Fetus birth weight above 4000g

### Clinical characteristics in women with and without PROM in the GDM group

The patients with GDM were divided into PROM and non-PROM groups, and the baseline data for the two subgroups are shown in Table 2. There were significant differences in age (*P* = 0.030) and uric acid (*P* = 0.030) between the two groups. There were no significant differences in uric acid between the GDM and non-GDM groups when PROM status was not considered (*P* = 0.130) (Table 1).

**Table 2:** Anthropometric parameters and biochemical indexes in the PROM group and non-PROM sub-groups in the GDM group

Variable	non-PROM group	PROM group	***P***-value
	(***n*** = 130)	(***n*** = 22)	
Age (years)	29 (26, 32)	32 (28, 34)	0.030*
Pre-pregnancy BMI (kg/m^2^)	22.21 (20.20, 24.72)	22.57 (20.22, 24.45)	0.778
SBP (mmHg)	114.00 (106.50, 120.00)	112.00 (100.00, 121.25)	0.676
DBP (mmHg)	70.00 (65.00, 80.00)	70.00 (65.25, 75.00)	0.478
HbA1c (%)	4.90 (4.30, 5.50)	4.90 (4.70, 5.10)	0.839
FPG (mmol/L)	4.80 (4.30, 5.50)	4.80 (4.50, 5.40)	0.746
1-h PG (mmol/L)	9.80 (8.10, 10.90)	10.10 (7.90, 10.90)	0.676
2-h PG (mmol/L)	8.30 (6.15, 9.15)	9.05 (6.98, 9.93)	0.140
FINS (mU/L)	10.00 (6.64, 13.03)	11.00 (5.30, 21.00)	0.494
1-h INS (mU/L)	76.97 (47.80, 119.28)	72.15 (40.50, 87.80)	0.444
2-h INS (mU/L)	81.30 (51.88, 130.30)	77.00 (49.41, 122.35)	0.579
ALT (U/L)	12 (10, 21)	11 (9, 14)	0.086
Total bilirubin (μmol/L)	7.70 (6.20, 9.60)	8.00 (6.00, 10.50)	0.923
Creatinine (umolL)	46 (43, 51)	49 (41, 52)	0.668
Uric acid (μmol/L)	233 (203, 255)	197 (187, 237)	0.030*
Triglycerides (mmol/L)	2.41 (1.90, 3.05)	2.84 (2.03, 4.04)	0.146
Total cholesterol (mmol/L)	5.95 (5.24, 6.59)	6.03 (5.44, 7.28)	0.353
White blood cell (×109/L)	9.98 ± 2.11	10.04 ± 2.39	0.974
Platelet count (×109/L)	226.96 ± 47.22	210.07 ± 48.49	0.155
Weight gain (Kg)	14.50 ± 6.50	12.78 ± 5.34	0.348
Fetus birth weight (g)	3545.95 ± 440.31	3510.26 ± 514.52	0.859

Data were expressed as median (interquartile range) or Mean ± standard deviation, Significant at *P* < 0.05. *P* < 0.05*

*PROM* premature rupture of the membranes, *BMI* body mass index*, HbA1c* hemoglobin A1C*, FPG* fasting plasma glucose*, 1-h PG* 1-hour postprandial glucose*, 2-h PG* 2-hour postprandial glucose*, FINS* fasting insulin, *1-h INS* 1-hour postprandial insulin*, 2-h INS* 2-hour postprandial insulin*, ALT* alanine aminotransferase

### Correlation between SUA and PROM

Binary logistic regression was conducted to assess the association of PROM with clinical variables with PROM as the dependent variable, as shown in Table 3. When the clinical variables (age, pre-pregnancy BMI, 1-h PG, parity, weight gain) that might affect outcomes were adjusted, uric acid (*P* = 0.043) was related to PROM in the GDM group in multivariate analysis. Furthermore, the uric acid of the whole population was stratified by three quantiles, and the results showed that the incidence of PROM decreased with the increase in blood uric acid tertiles. The corresponding ORs (95% CIs) were 0.13 [0.02–0.95] in the second tertile and 0.07 [0.01–0.93] in the upper tertile in comparison to the lower tertile (all *P* < 0.05).

**Table 3:** Multivariate logistic analysis of factors related to PROM in the non-GDM and GDM group

Variables	non-GDM		GDM	
	OR (95% CI)	***P***-value	OR (95% CI)	***P***-value
Age (years)	1.02 (0.84-1.22)	0.901	1.19 (1.01-1.42)	0.049*
Pre-pregnancy BMI (kg/m^2^)	1.01 (0.77-1.30)	0.984	1.18 (0.84-1.66)	0.345
1-h PG (mmol/L)	1.17 (0.71-1.91)	0.544	1.01 (0.66-1.54)	0.963
Parity	0.75 (0.18-3.15)	0.689	0.35 (0.05-2.23)	0.265
Weight gain (kg)	0.94 (0.84-1.06)	0.331	0.93 (0.79-1.09)	0.344
Uric acid levels (μmol/L)		0.579		0.043*
SUA tertiles (μmol/L)				
0-197	1		1	
198-247	2.53 (0.36-17.90)	0.352	0.13 (0.02-0.95)	0.044*
≥ 247	2.34 (0.43-12.84)	0.329	0.07 (0.01-0.93)	0.044*

*OR* odds ratio, *CI* confidence interval*;* Significant at *P* < 0.05. *P* < 0.05*

**Fig. 1:**
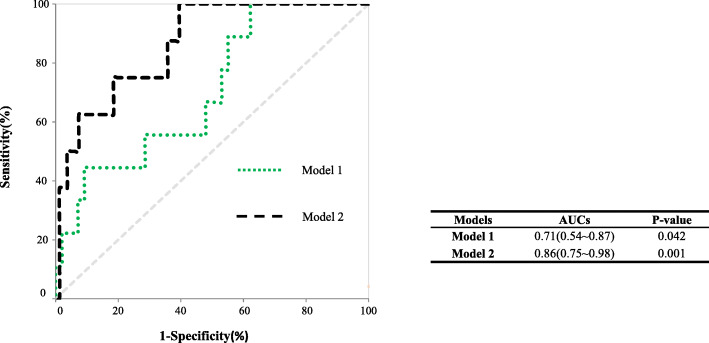
ROC curves for SUA predictive of PROM in the GDM group. Legend: Model 1: general clinical characteristic model 1 (age, pre-pregnancy BMI, 1-h PG, parity, weight gain); Model 2: uric acid is added as variable to the model

Furthermore, the predictive value of different models for PROM was analysed using receiver operating characteristic (ROC) analysis. As shown in Fig. 1, ROC curve analysis showed that the area under the curve of the general clinical characteristic model 1 was 0.71 [0.54–0.87], *P* < 0.05. When uric acid was added as a variable, the area under the curve in model 2 reached 0.86 [0.75–0.98], *P* < 0.001.

## Discussion

The present study indicated that women with PROM had lower levels of SUA than those without PROM, and the prevalence of PROM decreased with the increase in SUA tertiles among the GDM group. Additionally, multiple logistic regression analysis indicated that SUA was independently associated with PROM in the GDM group. Finally, the AUC was larger in the model incorporating SUA combined with general clinical parameters than that only considering general indices.

### Known risk factors affecting PROM

What we already know about PROM focuses on infectious and external factors, such as genital infections by chlamydia and/or gonorrhoea, intrauterine infection and cervical abnormalities before pregnancy [13]. Studies that have looked for metabolic factors related to PROM in pregnant women are rare. One study from a hospital in Ethiopia showed that a history of abortion, history of PROM, history of caesarean section, and abnormal vaginal discharge were positively associated with PROM [1]. A Chinese study suggested that migration as a result of urbanization, high rates of induced abortion, and preterm birth are potential risk factors for preterm PROM [14]. A Swedish study of PROM at or near term found no obvious risk factors and concluded that PROM close to term can be difficult to manage [15]. GDM is associated with adverse pregnancy outcomes, including PROM [7]. A study from Pennsylvania found that cord blood uric acid was higher in preterm PROM vs. preterm birth, but maternal plasma uric acid and glucose tolerance state were not considered [16]. To the best of our knowledge, the relationship of uric acid levels with PROM in women with GDM has not been studied previously. No model for individual prediction of PROM risk has been validated or used in clinical practice. By distinguishing between GDM and women with normal glucose tolerance, we identified SUA as an independent factor related to PROM for women with GDM. Of utmost importance is that as the concentration of uric acid increases, the risk of PROM decreases in the patients with GDM, and elevated SUA levels suggest a lower risk incidence of PROM after adjustment for other confounders.

### The importance of reasonable uric acid levels in GDM

The concentration of serum uric acid has two sides in the pathophysiological process of many diseases. Hyperglycaemia accelerates purine nucleotide synthesis, which in turn stimulates nucleotide breakdown and increases the concentration of nucleotide degradation products, including superoxide molecules and uric acid [17]. These substances directly damage islet function [18] and worsen diabetes complications [19]. However, uric acid is an antioxidant that can protect cells from OS damage. The relationship between uric acid and cardiovascular event rates is J-shaped [20]. High SUA levels due to uric acid toxicity increase cardiovascular events, but when SUA levels are low, cardiovascular events are also increased; this analogous finding was observed in patients with vasospastic angina [21]. The effects of SUA are also the same in acute ischaemic stroke patients [22].

Reference values for pregnancy can be extremely important for making clinical decisions. Normal reference values are usually based on blood samples from nonpregnant women. However, uric acid levels are higher in women late in pregnancy than in nonpregnant women [23]. Additionally, hyperglycaemia increases oxidative stress products; hence SUA levels should also be increased in GDM patients. Women with GDM should have higher SUA levels in late pregnancy and in hyperglycaemic states. However, when the SUA levels do not increase correspondingly, women with GDM are more prone to PROM

### The possible mechanism of uric acid, oxidative stress and PROM

A general health screening study of individuals revealed that SUA levels are positively associated with antioxidant potential; these results indicate that SUA can prevent oxidative damage [24]. GDM is characterized by increased levels of OS biomarkers. Placental histological examinations have indicated that OS plays an important role in inducing inflammation and impairing perfusion in the placenta [10]. Our study showed that SUA levels were inversely correlated with PROM in GDM subjects. OS damage is also an important factor in the initiation of cell apoptosis. GDM and type 2 diabetes mellitus (T2DM) subjects have increased expression levels of base excision repair proteins, and hyperglycaemia levels are associated with apoptosis [25]. Proteomics research confirmed by western blot and RT-PCR analyses revealed that placental tissues from PROM subjects had lower levels of the anti-apoptotic protein HSPA2 and higher expression levels of PRDX3 and annexin A1 (associated with apoptosis) than placental tissues from normal pregnancy subjects [26]. The increase in apoptosis caused by the decrease in antioxidant stress may be another important cause of PROM. We hypothesized that the antioxidant capacity of relatively low SUA levels cannot exert its usual protective effects; thus, women with low SUA are at risk of PROM. This study indicates that maternal age is another important factor affecting PROM, as it was significantly higher in GDM patients with PROM. This is consistent with the Chinese study of risk factors for PROM [14]. The percentage decrease in enzymatic antioxidants and antioxidant vitamins with age is significant [27]. In this study, when divided the subjects into two groups by age ≤ 29 years and age > 29 years old, the data showed that SUA was only associated with PROM in elderly pregnant women (age > 29 years old) in the GDM group (Supplemental Table 1). The protective effect of relatively high SUA levels on PROM may be more important in elderly women with GDM. After the implementation of the nationwide two-child policy in China, the number of elderly pregnant women increased, which may be another important factor in the association with adverse pregnancy outcomes.

There was some strength in this study. Blood biochemical tests (including SUA) were collected from all subjects at 24-28th weeks of gestation to exclude the effects of differences in gestational weeks on the results. All the subjects were women because of pregnancy, and none of the subjects consumed alcohol. Consequently, possible SUA-influencing factors, such as sex and alcohol consumption, were excluded. Except for the factors that may affect glucose metabolism, there were no other recruitment restrictions, thus the study population should be a good representation. There are some limitations to this study. This was a single centre study, so the number of subjects was limited. The follow-up period was short; therefore we could make no conclusions on the long-term pregnancy outcomes. The related uric acid metabolism pathways also need further study.

## Conclusion

In conclusion, changes in uric acid levels at physiological concentrations can have adverse effects on PROM, and maternal age is another important factor affecting only pregnant women with gestational diabetes. The moderate increase in uric acid levels at 24-28th gestation may have a potential protective effect on PROM with GDM. At present, we know that the concentration of uric acid is different in different genders and physiological states, and the influence of uric acid on disease outcomes in different diseases is worthy of further study.

## Supplementary Information

**Additional file 1: Supplemental Table 1**. Multivariate logistic analysis of factors related to PROM at different ages in two groups

## Data Availability

The datasets used and/or analysed during the current study are available from the corresponding author on reasonable request. The data contained potentially identifying and sensitive patient information as well as the consent agreement supervised by the ethics committee. Data are available from the Ethics Committee of Central Hospital of Shanghai Fengxian District for researchers who meet the criteria for access to confidential data.

## Abbreviations

SUA: Serum uric acid
PROM: Premature rupture of membranes
IADPSG: International Association of Diabetes and Pregnancy Study Groups
GDM: Gestational diabetes
BMI: Body mass index
OS: Oxidative stress
FBG: Fasting blood glucose
HbA1c: Glycosylated haemoglobin
1-h PG: 1-h postprandial glucose
2-h PG: 2-h postprandial glucose
FINS: Fasting insulin
1-h INS: 1-h postprandial insulin
2-h INS: 2-h postprandial insulin
ALT: Alanine aminotransferase
