# Supplementary material for: The protective role of serum uric acid against premature membrane rupture in gestational diabetes: a cross-sectional study
Source: BMC Endocr Disord. 2021 May 6;21:95. doi: 10.1186/s12902-021-00736-3 (PMC8101033; doi:10.1186/s12902-021-00736-3)
Supplement: Supplementary file 1 — Additional file 1: Supplemental Table 1. Multivariate logistic analysis of factors related to PROM at different ages in two groups [file 12902_2021_736_MOESM1_ESM.docx]

Supplementary Materials 1

Table S1 Multivariate logistic analysis of factors related to PROM in different age in two groups

|  | | | OR(95%CI) | | P-value |
| --- | --- | --- | --- | --- | --- |
|  |  |  |  |  |  |
| non-GDM group | Youger Pregnant | Pre-pregnancy BMI(kg/m^2)^ | 0.7263-1.3503 | | 0.9510 |
|  |  | UA | 0.9941-1.0137 | | 0.4415 |
|  |  | 1-h PG(mmol/L) | 0.581-2.1258 | | 0.7496 |
|  |  | Parity | 0.1557-5.1603 | | 0.9026 |
|  |  | Weight gain（Kg） | 0.8402-1.1437 | | 0.7999 |
|  | Older Pregnant | Pre-pregnancy BMI(kg/m^2^) | 0.8106-2.3338 | | 0.2374 |
|  |  | UA | 0.979-1.0072 | | 0.3304 |
|  |  | 1-h PG(mmol/L) | 0.5166-2.9887 | | 0.6276 |
|  |  | Parity | 0.0173-2.4264 | | 0.2087 |
|  |  | Weight gain（Kg） | 0.6707-1.0414 | | 0.1100 |
| GDM group | Youger Pregnant | Pre-pregnancy BMI(kg/m^2^) | 0.8218-2.3407 | | 0.2206 |
|  |  | UA | 0.9249-1.0147 | | 0.1794 |
|  |  | 1-h PG(mmol/L) | 0.3898-1.6521 | | 0.5504 |
|  |  | Parity | 0.1107-25.3037 | | 0.7101 |
|  |  | Weight gain（Kg） | 0.7265-1.2519 | | 0.7329 |
|  | Older Pregnant | Pre-pregnancy BMI(kg/m^2^) | 0.6724-3.4508 | | 0.3132 |
|  |  | UA | 0.8493-0.9999* | | 0.0499***** |
|  |  | 1-h PG(mmol/L) | 0.5871-2.0917 | | 0.7513 |
|  |  | Parity | 0.0004-2.7391 | | 0.1318 |
|  |  | Weight gain（Kg） | 0.7866-1.1735 | | 0.6946 |
| *OR:* odds ratio; *CI*: confidence interval; Significant at P<0.05. P<0.05***** | | | | | |
| *1-h PG*: 1-hour postprandial glucose，*UA：*uric acid | | | |  | |
